# Advanced small cell carcinoma of the bladder: clinical characteristics, treatment patterns and outcomes in 960 patients and comparison with urothelial carcinoma

**DOI:** 10.1002/cam4.577

**Published:** 2015-12-18

**Authors:** Daniel M. Geynisman, Elizabeth Handorf, Yu‐Ning Wong, Jamie Doyle, Elizabeth R. Plimack, Eric M. Horwitz, Daniel J. Canter, Robert G. Uzzo, Alexander Kutikov, Marc C. Smaldone

**Affiliations:** ^1^ Department of Medical OncologyFox Chase Cancer Center‐Temple University Health SystemPhiladelphiaPennsylvania; ^2^ Biostatistics & Bioinformatics FacilityFox Chase Cancer Center‐Temple University Health SystemPhiladelphiaPennsylvania; ^3^ Department of Radiation OncologyFox Chase Cancer Center‐Temple University Health SystemPhiladelphiaPennsylvania; ^4^ The Fox Chase Cancer CenterEinstein Health Network and The Urologic Institute of Southeastern PennsylvaniaPhiladelphiaPennsylvania; ^5^ Division of Urologic OncologyDepartment of Surgical OncologyFox Chase Cancer Center‐Temple University Health SystemPhiladelphiaPennsylvania

**Keywords:** Bladder cancer, outcomes, small cell bladder cancer, treatment patterns, urothelial carcinoma

## Abstract

To describe the clinical characteristics, treatment patterns and outcomes in advanced small cell bladder cancer (aSCBC) patients and compare to those with urothelial carcinoma (UC). Individuals in the National Cancer Data Base with a diagnosis of either nodal (TxN+M0) or distant metastatic (TxNxM1) disease were identified from 1998 to 2010. We assessed the relationships between stage, treatment modalities and survival in the aSCBC cohort and compared these to UC patients. In the 960 patient aSCBC cohort (62% M1), 50% received palliative therapy alone, 68% in M1 versus 21% in M0 groups (*P *<* *0.0001). Single modality local therapy (15%) and surgical (21%) or radiation‐based (14%) multimodal therapy (MMT) were used in the other 50%. Cystectomy‐based MMT was utilized in 45% of N+M0 versus 6.4% of NxM1 patients (*P *<* *0.0001). Median overall survival (OS) for aSCBC patients was 8.6 months; 13.0 months in N+M0 versus 5.3 months in NxM1 patients (*P *<* *0.0001). Survival was similar between TxN1M0 and TxN2‐3M0 patients (14.8 months vs. 12.1 months, *P *=* *0.15). Urothelial carcinoma patients (*n *=* *27,796, 45% M1) lived longer compared to aSCBC patients in the N+M0 group (17.3 months vs. 13.0 months, *P *=* *0.0007). There were not clinically significant differences in OS between UC and aSCBC patients in the M1 group. Advanced SCBC is a rare disease with a poor survival and palliative therapy is common, especially in M1 patients. In comparison to UC, the outcomes for aSCBC patients are worse in those with lymph node only involvement but similar in those with distant disease.

## Introduction

In 2015 in the United States, an estimated 74,000 people will be newly diagnosed with bladder cancer and almost 16,000 people will die from this disease 1. Worldwide, bladder cancer causes approximately 150,000 deaths per year and is the most common genitourinary malignancy after prostate cancer 2. In the United States and Europe, the majority of bladder cancer cases (~90%) are of the urothelial carcinoma (UC) variety, but a number of less common subtypes exist 3. Small cell bladder cancer (SCBC), originally identified in 1981, is a rare and aggressive subtype of bladder cancer, is frequently admixed with other histologic subtypes, but may arise from the same clonal population as UC 4, 5, 6, 7. Most frequently presenting with hematuria, SCBC has been found in <1% of bladder cancer patients and often mimics the behavior of small cell lung carcinoma—rapid progression, early metastasis, and high mortality rate 8, 9.

A Surveillance, Epidemiology, and End Results database (SEER) analysis of 642 SCBC patients from 1991 to 2005 showed a rise in incidence of SCBC from 0.3% to 0.6% with approximately 500 new cases per year, a 3:1 male to female ratio and a median age at presentation of 73 years. Of these, 50% presented with American Joint Committee on Cancer (AJCC) Stage III or IV disease 10 (36% stage IV) and the median overall survival (OS) for the entire cohort was 11 months 11. Because of its rarity, standardized treatment algorithms for SCBC have not been identified and strategies have been adapted from the treatment of small cell lung cancer, utilizing a multimodal approach of chemotherapy, radiation therapy, and surgery 12. In both localized and advanced disease, no randomized prospective trials have been conducted and most data comes from single center retrospective experiences or literature reviews 8, 13, 14, 15, 16, 17, 18, 19, 20. Furthermore, there is limited data regarding contemporary population‐level practice patterns.

The aim of this study was to utilize the National Cancer Data Base (NCDB) in order to report the clinical characteristics, treatment patterns and outcomes in AJCC Stage IV (node‐positive and/or with distant metastatic disease) and hereafter defined as “advanced” SCBC (aSCBC) patients, and to compare those outcomes to those with advanced UC.

## Patients and Methods

### Cohort definition

A program of the American College of Surgeons, Commission on Cancer, and American Cancer Society, the NCDB is a national cancer registry that was established in 1989 and serves as a comprehensive clinical surveillance resource for cancer care in the United States. The NCDB compiles data from more than 1500 commission‐accredited cancer programs in the United States and Puerto Rico, and captures approximately to 70% of all newly diagnosed cancer cases 21.

Our analytic cohort was restricted to individuals aged 18 or older with a diagnosis of either SCBC (*International Classification of Diseases for Oncology, 3rd Edition* [*ICD‐O‐3*] histology site codes 8002, 8041, 8042, 8043, 8044, 8045, 8246) or UC of the bladder (*ICD‐O‐3* histology site codes 8120 and 8130), restricted to those with either a clinical or pathologic staging of N > 0 and/or M > 0 or both from 1998 to 2010. The NCDB provides files specific to each organ site, and only bladder files were used for these analysis. The codes used were consistent with previous studies in SCBC 11, 22. Patient socioeconomic characteristics were provided using census tract data. Comorbidity burden was determined using the Charlson–Deyo classification and categorized as 0, 1, or ≥2. Vital status to determine trends in OS was only available for patients identified prior to 2006.

### Statistical analyzes

We grouped patients as lymph node positive and without distant metastatic disease (TxN+M0, [where “N” can = 1–3]), or with distant metastatic disease (TxNxM1, [where “N” can = 0–3]), and tabulated groups by patient characteristics and type of treatment received. We assessed the relationships between TNM stage and patient characteristics as well as treatment modalities employed, using Chi‐squared tests. For treatment modality comparisons, we focused on overall differences in treatment trends between the two TNM groups, thus obtaining an overall significance value. We then compared treatment rates among SCBC versus UC patients, also using Chi‐squared tests. In the subset of patients where survival data was available (diagnosis between 1998 and 2005), we evaluated survival within and between the aSCBC and UC cohorts, both overall and by TNM status. Survival curves were constructed using the method of Kaplan and Meier, and we tested for differences in survival using log‐rank tests. Multivariate analysis using Cox proportional hazards regression was performed when comparing UC to aSCBC patients with TxN+M0 disease, adjusting for age, race and sex. Charlson–Deyo Score was available for patients diagnosed in years 2003–2005; we therefore ran a separate model adjusting for Charlson score in this subgroup. All statistical analyzes were performed using SAS software (version 9.3, SAS Institute Inc., Cary, NC, USA) or Stata (version 12.1, StataCorp LP College Station, TX, USA) with *P* < 0.05 meeting statistical significance.

## Results

### Patient characteristics

From 1998 to 2010, there were 3329 patients identified with aSCBC, of whom 960 (28.8%) were N > 0 or M > 0 at diagnosis (mean age 69.2, range 29–90, 75.4% male, 62% with M > 0 disease). Patient characteristics are shown in Table 1. Over 75% of aSCBC patients were >60 years of age at diagnosis, 71% had a Charlson–Deyo Score of 0, 75% were male and 92% were white. We noted statistically significant differences in age, tumor type and facility type (*P* < 0.01) between the TxNxM1 and TxN+M0 groups. This advanced SCBC patient cohort was compared with regard to survival to 27,796 patients with UC who were N > 0 or M > 0 or both at the time of diagnosis (mean age 68.9, range 19–90).

**Table 1:** Advanced small cell bladder cancer and urothelial carcinoma patient characteristics by TNM status

	Overall			Small cell		
	Urothelial	Small cell	*P* value^a^	TxN+M‐	TxNxM1	*P* value^b^
*N*	27,796	960		364 (38%)	596 (62%)	
Age (years), *N* (%)						
<50	1663 (6.0)	65 (6.8)	0.6672	27 (41.5)	38 (58.5)	0.0076
≥50–60	4381 (15.8)	144 (15.0)		68 (47.2)	76 (52.8)	
>60–70	7409 (26.7)	249 (25.9)		103 (41.4)	146 (58.6)	
≥70	14,343 (51.6)	502 (52.3)		166 (33.1)	336 (66.9)	
Charlson–Deyo Score^c^, *N* (%)						
0	13,251 (72.0)	505 (70.7)	0.4175	173 (34.3)	332 (65.7)	0.6646
1	3826 (20.8)	162 (22.7)		61 (37.7)	101 (62.3)	
≥2	1331 (7.2)	47 (6.6)		15 (31.9)	32 (68.1)	
Gender, *N* (%)						
Male	19,650 (70.7)	724 (75.4)	0.0015	281 (38.8)	443 (61.2)	0.3165
Female	8146 (29.3)	236 (24.6)		83 (35.2)	153 (64.8)	
Race, *N* (%)						
White	24,834 (89.3)	881 (91.8)	0.0529	336 (38.1)	545 (61.9)	0.7555
AA	2159 (7.8)	59 (6.1)		22 (37.3)	37 (62.7)	
Other/unknown	803 (2.9)	20 (2.1)		6 (30.0)	14 (70.0)	
Median income, *N* (%)						
<$30K	3657 (13.2)	116 (12.1)	0.4249	47 (40.5)	69 (59.5)	0.8488
$30–34.9K	5080 (18.3)	160 (16.7)		55 (34.4)	105 (65.6)	
$35–44.9K	7745 (27.9)	277 (28.9)		104 (37.5)	173 (62.5)	
>$45K	9903 (35.6)	362 (37.7)		140 (38.7)	222 (61.3)	
Unknown	1411 (5.1)	45 (4.7)		18 (40.0)	27 (60.0)	
Facility type, *N* (%)						
Community	3948 (14.2)	138 (14.4)	0.4394	43 (31.2)	95 (68.8)	0.0002
Comprehensive community	12,715 (45.7)	452 (47.1)		150 (33.2)	302 (66.8)	
Academic	10,689 (38.5)	350 (36.5)		165 (47.1)	185 (52.9)	
Unknown	444 (1.6)	20 (2.1)		6 (30.0)	14 (70.0)	

a *P* value reflects the overall comparison between urothelial and small cell carcinoma patients.

b *P* value reflects comparison between TxN+M‐ and TxNxM1 small cell carcinoma patients.

c A total of 9388 Charlson–Deyo Scores were missing for urothelial carcinoma patients and 246 Charlson–Deyo Scores were missing for small cell carcinoma patients.

### Treatment distribution

**Figure 1:**
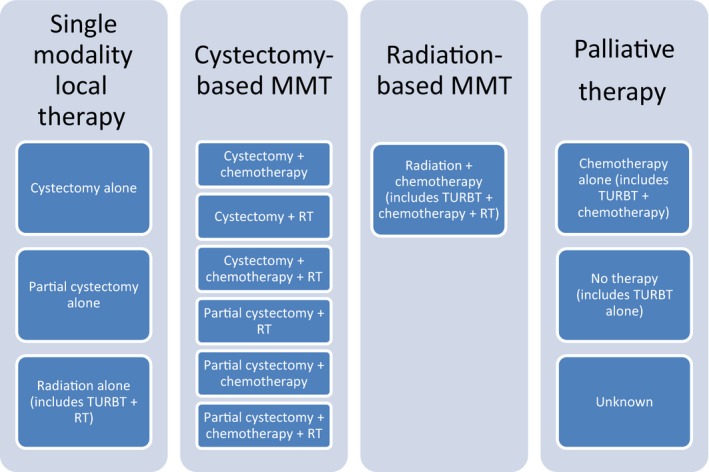
Classification of subjects based on therapy administered. MMT, multimodal; RT, radiation therapy; TURBT, transurethral resection of bladder tumor. Of note, classification does not denote original therapeutic intent (palliative vs. curative) but rather the actual treatment received.

We initially categorized treatment modalities into 13 separate categories and then collapsed them into four groups that mimic clinical practice: single modality local therapy, cystectomy‐based multimodal therapy, radiation‐based multimodal therapy, and palliative therapy (Fig. 1). Importantly, TURBT was not considered a definitive treatment modality by itself as given the aggressive nature of aSCBC, TURBT alone is rarely curative, but rather considered either as part of another treatment (e.g., radiation plus TURBT) or if TURBT was truly the only treatment performed, as “no treatment” under the “palliative therapy” group. Also, palliative therapy was defined as either a lack of any therapy or chemotherapy alone given that the later is not considered curative as single modality in SCBC. Treatment modalities used in the aSCBC group overall and by TNM status are presented in Table 2 and demonstrate significant differences between the two TNM sub‐groups (*P *<* *0.0001). For the entire aSCBC cohort, 50% of subjects received palliative therapy alone, of which 20% received no therapy beyond a TURBT. The other half was split between single modality local therapy (15%) and surgical (21%) or radiation‐based (14%) multimodal therapy. Significant differences in treatment approaches were seen between N+M0 and NxM1 groups. For example, 68% of patients with metastatic disease received palliative therapy only compared to 21% in the M0 group (*P* < 0.0001) and cystectomy based MMT was utilized in 45% of N+M0 versus 6.4% of NxM1 patients (*P* < 0.0001). Approximately 12–15% of patients in both groups received radiation‐based MMT. Almost 28% of NxM1 patients received no further therapy after their diagnosis compared to 7% of N+M0 patients.

**Table 2:** Treatment modalities utilized in aSCBC patients by TNM status (*P* < 0.0001)

	Overall	TxN+M0	TxNxM1
*N*	960	364	596
	*N* (%)	*N* (%)	N (%)
Single modality local therapy	141 (14.7)	78 (21.4)	63 (10.6)
Cystectomy alone	84 (8.8)	61 (16.8)	23 (3.9)
Partial cystectomy alone	6 (0.6)	3 (0.8)	3 (0.5)
Radiation alone^a^	51 (5.3)	14 (3.8)	37 (6.2)
Palliative therapy	483 (50.3)	76 (20.9)	407 (68.3)
Chemotherapy alone^b^	292 (30.4)	51 (14.0)	241 (40.4)
No therapy c	190 (19.8)	25 (6.9)	165 (27.7)
Unknown	1 (0.1)	0 (0)	1 (0.2)
Cystectomy‐based MMT^d^	203 (21.1)	165 (45.3)	38 (6.4)
Cystectomy + radiation	6 (0.6)	2 (0.5)	4 (0.7)
Cystectomy + chemotherapy	145 (15.1)	126 (34.6)	19 (3.2)
Cystectomy + chemotherapy + radiation	22 (2.3)	19 (5.2)	3 (0.5)
Partial cystectomy + radiation	1 (0.1)	1 (0.3)	0 (0)
Partial cystectomy + chemotherapy	14 (1.5)	8 (2.2)	6 (1.0)
Partial cystectomy + chemotherapy + radiation	15 (1.6)	9 (2.5)	6 (1.0)
Radiation‐based MMT^e^	133 (13.9)	45 (12.4)	88 (14.8)

a Radiation alone includes TURBT + radiation.

b Chemotherapy alone includes TURBT + chemotherapy.

c No therapy includes TURBT alone.

d MMT‐multimodal treatment.

e Includes radiation + chemotherapy or radiation + TURBT + chemotherapy.

In Table 3, we compare treatment modalities used in SCBC patients to those used in UC patients that are comparable by TNM stage. Differences were noted within each TNM strata depending on histologic type. In the N+M0 patients single modality therapy was used more frequently in UC (36.8% vs. 21.4%) and cystectomy‐based MMT was more common in SCBC (45.3% vs. 38.5%); radiation‐based MMT was more common as the definitive approach in aSCBC than UC in both groups (12.4% vs. 5.4% in N+M0; 14.8% vs. 11.9% in NxM1) (*P *<* *0.0001). The rate of palliative therapy alone was the same for UC and aSCBC in the N+M0 group (19.4% vs. 20.9%, respectively) and only slightly different in the M1 group (63.0% UC vs. 68.3% aSCBC).

**Table 3:** Treatment modalities utilized in patients with aSCBC versus urothelial bladder cancer (*P* < 0.0001)

	TxN+M0			TxNxM1		
	Overall	Small cell	Urothelial	Overall	Small cell	Urothelial
*N*	15,651	364	15,287	13,105	596	12,509
Single modality local therapy, *N* (%)	5703 (36.4)	78 (21.4)	5625 (36.8)	2312 (17.6)	63 (10.6)	2249 (18.0)
Cystectomy alone	5168 (33.0)	61 (16.8)	5107 (33.4)	783 (6.0)	23 (3.9)	760 (6.1)
Partial cystectomy alone	127 (0.8)	3 (0.8)	124 (0.8)	88 (0.7)	3 (0.5)	85 (0.7)
Radiation alone^a^	408 (2.6)	14 (3.8)	394 (2.6)	1441 (11.0)	37 (6.2)	1404 (11.2)
Palliative, *N* (%)	3039 (19.4)	76 (20.9)	2963 (19.4)	8283 (63.2)	407 (68.3)	7876 (63.0)
Chemotherapy alone^b^	1571 (10.0)	51 (14.0)	1520 (9.9)	3621 (27.6)	241 (40.4)	3380 (27.0)
No therapy c	1456 (9.3)	25 (6.9)	1431 (9.4)	4628 (35.3)	165 (27.7)	4463 (35.7)
Unknown	12 (0.1)	0 (0)	12 (0.1)	34 (0.2)	1 (0.2)	33 (0.3)
Cystectomy‐based MMT^d^, *N* (%)	6044 (38.6)	165 (45.3)	5879 (38.5)	937 (7.1)	38 (6.4)	899 (7.2)
Cystectomy + radiation	149 (1.0)	2 (0.5)	147 (1.0)	108 (0.8)	4 (0.7)	104 (0.8)
Cystectomy + chemotherapy	5198 (33.2)	126 (34.6)	5072 (33.2)	596 (4.5)	19 (3.2)	577 (4.6)
Cystectomy + chemotherapy + radiation	484 (3.1)	19 (5.2)	465 (3.0)	145 (1.1)	3 (0.5)	142 (1.1)
Partial cystectomy + radiation	17 (0.1)	1 (0.3)	16 (0.1)	14 (0.1)	0 (0)	14 (0.1)
Partial cystectomy + chemotherapy	144 (0.9)	8 (2.2)	136 (0.9)	48 (0.4)	6 (1.0)	42 (0.3)
Partial cystectomy + chemotherapy + radiation	52 (0.3)	9 (2.5)	43 (0.3)	26 (0.2)	6 (1.0)	20 (0.2)
Radiation‐based MMT^e^	865 (5.5)	45 (12.4)	820 (5.4)	1573 (12.0)	88 (14.8)	1485 (11.9)

a Radiation alone includes TURBT + radiation.

b Chemotherapy alone includes TURBT + chemotherapy.

c No therapy includes TURBT alone.

d MMT‐multimodal treatment.

e Includes radiation + chemotherapy or radiation + TURBT + chemotherapy.

### Overall survival

**Figure 2:**
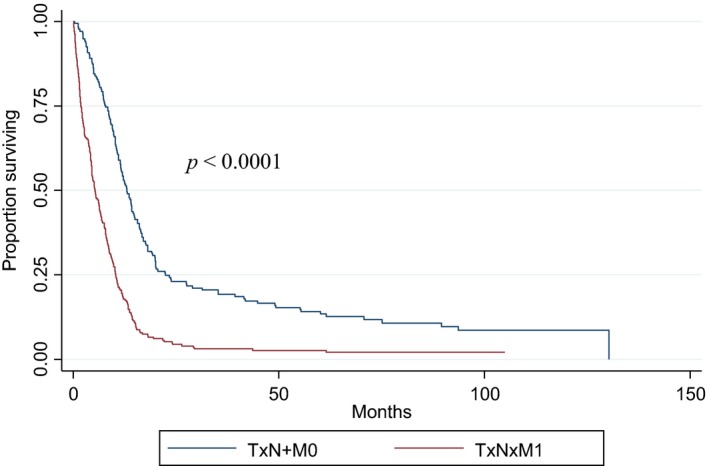
Overall survival of small cell bladder patients by TNM status. Median OS of TxN+M0 patients was 13.04 months (CI: 11.53–14.78) versus 5.29 months (CI: 4.5–6.41) for TxNxM1 patients. *P* < 0.0001.

**Figure 3:**
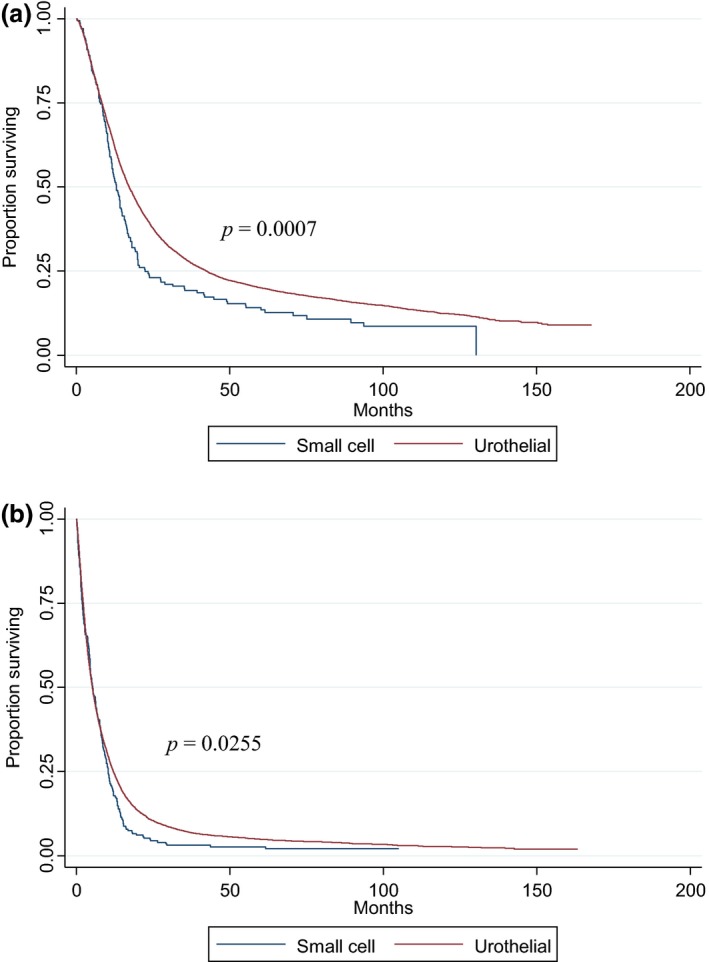
(A) Overall survival (OS) of TxN+M0 small cell versus urothelial bladder cancer patients. Median OS of small cell patients 13.04 months (CI: 11.53–14.78) versus 17.25 months (CI: 16.66–17.81) for urothelial carcinoma (UC) patients. *P* = 0.0007. (B) OS of TxNxM1 small cell versus urothelial bladder cancer patients. Median OS of small cell patients 5.29 months (CI: 4.5–6.67) versus 5.19 months for UC patients (CI: 4.99–5.42). *P* = 0.0255.

**Figure 4:**
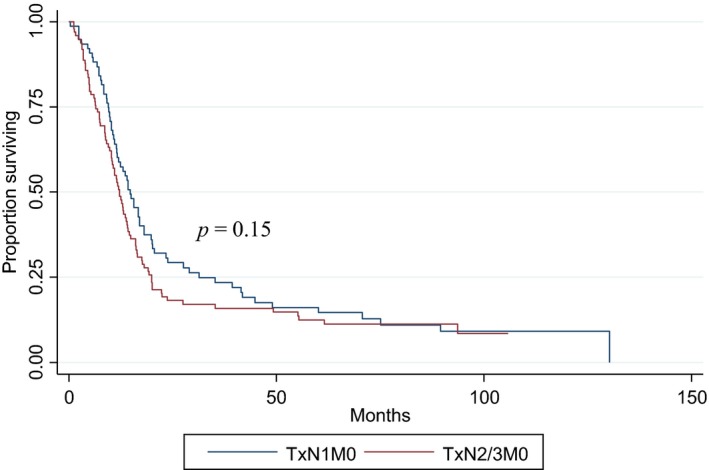
The Kaplan–Meier survival estimate for TxN1M0 versus TxN2M0 or TxN3M0. Median OS of TxN1M‐ patients 14.78 months (CI: 11.56–18.07) versus 12.12 months (CI: 10.25–14.03) for TxN2M‐ or TxN3M‐ patients. *P* = 0.15.

Median follow‐up for the 421 SCBC patients and 15,482 UC patients with survival data available (1998–2006) were 8.25 months (range: 0–130 months) and 10.05 months (range: 0–168 months), respectively. Median OS for the entire aSCBC cohort was 8.61 months; it was found to be significantly higher in the TxN+M0 group (13.04 months) than the TxNxM1 group (5.29 months) (*P* < 0.0001) (Fig. 2). UC patients had a longer median OS compared to aSCBC patients in the TxN+M0 group (17.25 months vs. 13.04 months respectively, *P *=* *0.0007), however in the TxNxM1 group UC patients had a shorter median OS compared to aSCBC patients (5.19 months vs. 5.29 months respectively, *P *=* *0.0255) (Fig. 3A and B). In multivariate analysis, age and sex were both significantly related to OS. After analysis was adjusted for these factors, in patients with TxN+M0 disease, histology maintained a statistically significant effect on OS (HR, 1.37; 95% CI, 1.17–1.61; *P* = 0.0001). In the subgroup of patients diagnosed between 2003 and 2005, the effect of aSCBC histology was stronger on unadjusted analysis (HR = 1.49, 95% CI 1.17–1.89, *P* = 0.001), but including comorbidities in the multivariate model did not result in appreciably different results (HR, 1.52; 95% CI, 1.21–1.92, *P* = 0.0004). When comparing OS by node status (N1 vs. N2‐3) in M0 aSCBC patients, no significant difference was noted (14.78 months vs. 12.12 months respectively, *P *=* *0.15) (Fig. 4).

## Discussion

Small cell bladder cancer is a rare subtype of bladder cancer with a poor prognosis and a lack of standardized treatment options. Approximately 40% of SCBC patients present with advanced disease at the time of diagnosis 11. Our analysis revealed that over 60% of advanced SCBC patients present with M1 disease, carrying with it a dismal prognosis with a median OS of less than 6 months. In the entire aSCBC cohort, over 50% received palliative therapy alone with 15% receiving single modality local therapy, 21% cystectomy‐based MMT and 14% radiation‐based MMT. Most patients were over 60 years of age, male, and white, similar to the previous SEER analysis 11.

Not unexpectedly, we found treatment approaches to be different between the N+M0 and NxM1 groups. In the M0 group, 21% got palliative therapy versus 68% in the M1 group and single modality therapy was used twice as frequently in N+M0 patients (21% vs. 11%). Cystectomy‐based MMT was used in almost half of N+ patients, but only 6% in those with metastatic disease. Radiation‐based MMT, an approach that has been advocated in the past for aSCBC patients, was used in less than 15% of all patients. Over 27% of M1 patients and almost 7% of M0 patients received no therapy beyond a TURBT, presumably in favor of supportive care alone. In respect to chemotherapy approaches, although our analysis is not able to capture the types of drugs prescribed, cisplatin with etoposide, much like for small cell lung cancer, remains the standard of care for those who are able to tolerate it. A variety of other regimens, including ifosfamide/doxorubicin, paclitaxel, carboplatin and irinotecan, has been used, albeit with limited success 12, 20.

Given the aggressive nature aSCBC, the extent of non‐multimodal therapy seen in our study may potentially reflect the lack of consensus regarding optimal treatment of this malignancy 19. Over 17% of node‐positive patients received a cystectomy as their only treatment as did over 4% of those with M1 disease. It is possible that it was originally planned for these patients to undergo further postsurgery therapy such as adjuvant chemotherapy but due to surgical complications or rapid progression of disease, they were never able to receive additional treatment.

A secondary analysis of 533 SCBC patients of any AJCC stage from the aforementioned SEER cohort for whom treatment information was available showed that 54% had a transurethral resection of bladder tumor (TURBT) as their only surgical intervention and less than 20% received a potentially curative regimen such as cystectomy with perioperative chemotherapy or TURBT followed by chemotherapy with radiation (5 year OS 26% vs. 19% respectively, *P *>* *0.05) 22. Further supporting the benefit of MMT, Patel et al. conducted a NCDB analysis of less advanced 625 SCBC patients from 1998 to 2010 with cTis‐T4N0M0 tumors of whom 82.8% had ≤ cT2 disease 23. In their cohort, 19% underwent a cystectomy of whom 60% also received either neoadjuvant or adjuvant chemotherapy, 27.8% received only a TURBT or partial cystectomy, and 53.3% received a TURBT and/or partial cystectomy with either chemotherapy and/or radiation. The 3 year OS rate was 33% and in multivariate analysis, when controlling for age, CCI and stage, bladder‐preservation therapy (defined as TURBT or partial cystectomy) combined with chemotherapy and/or radiation was associated with longer OS compared to bladder‐preservation therapy alone (HR 0.51 (0.39–0.69), *P* < 0.001). Furthermore, in those with localized SCBC several studies support the use of neoadjuvant chemotherapy to improve OS, again supporting a multimodal approach to this disease 19, 24.

The OS in our study is generally similar to previously published cohorts. For those with M1 disease, the median OS of 5.3 months is lower than usually reported with the exception of 30 Veterans Affairs Health System patients with extensive SCBC disease whose OS was preliminarily reported to be 4.2 months 16. A more encouraging example of 12 patients, of whom 7 had metastatic disease outside the lymph nodes, and who were treated with alternating ifosfamide/doxorubicin and etoposide/cisplatin, had a median OS of 13.3 months 24. These data underscore the lack of effective treatment options for this group of patients. Compared to UC patients, the OS of the aSCBC cohort, although statistically superior, was clinically the same, and thus our multivariate analysis focused on the TxN+M0 patients. Furthermore, comorbidity adjustment was only possible for 2 years, but given the lethality of the disease, most likely would make little difference on the overall outcome.

A two‐tier staging system for SCBC has been proposed to mimic what is used in small cell lung cancer. Patients may be categorized into limited (T1‐3N0‐1M0) versus extensive (TxNxM1 or T4NxMx or TxN2‐3M0) groups 11. Our SCBC cohort consisted mainly of extensive disease patients, only 176 (18.3% of the 960 patients) were N1M0, but when we analyzed our N+M0 subgroup, by separating them into N1 versus N2‐3 cohorts, we found no significant difference is OS, although our ability to detect such a difference may be limited by sample size (Fig. 4). Finally, we wanted to compare aSCBC patients to those with the much more common UC histology in order to evaluate differences in treatment patterns and outcomes. When compared to UC, aSCBC patients with N+M0 disease were less likely to undergo a cystectomy alone and more likely to receive radiation‐based MMT. This may be explained by an expectation of rapid disease progression on the part of the treatment team thus leading to a more conservative approach. However, only 228 patients from the entire cohort of 960 (23.75%) received radiation as part of their treatment (138/596 for M1 and 90/364 for M0), less than would be expected given the parallels with small cell lung cancer and previous small studies is SCBC supporting this approach 25, 26, 27, 28, 29, 30. In contrast, metastatic UC and aSCBC patients appear to be treated similarly, with, 63–68% receiving palliative therapy with the OS in our M1 UC group significantly lower than seen in large phase III trials, where those with visceral disease had a median OS of 10.3 months 31. This discrepancy is likely due to careful patient selection for clinical trials such that even those with M1 disease are relatively healthy with a good performance status.

There are inherent limitations to interpretations of our data including most importantly the retrospective nature of the data with a lack of randomization between treatment modalities. Therefore, we a priori decided not to compare OS based on treatment modalities, as this would have introduced selection bias that could not have been controlled for appropriately and limited our comparisons based on TNM stage and histology (aSCBC vs. UC). Also, there is limited survival follow‐up that does not go beyond 2005. Furthermore, there was no central pathologic review to confirm small cell histology or whether other histologic components were seen. In the NCDB, the administration of chemotherapy may not be completely captured given that some of the subjects may have received therapy at a non‐NCDB reporting hospital or practice 32, and the doses and schedule of chemotherapy administration is not recorded. However, only five patients were diagnosed at a particular reporting facility, but received all subsequent treatments elsewhere. Finally, while only generalizable to hospitals reporting to the NCDB, our analysis's major strength is the large number of patients with advanced disease compared to prior reports.

In conclusion, the utilization of treatment modalities vary significantly depending on TNM status even within this advanced SCBC patient group. The overall prognosis of aSCBC is poor and palliative therapy is common. Those with lymph node only involvement live significantly longer, and may be the most appropriate candidates for consideration of aggressive MMT therapy. Given that SCBC is a rare disease, a comprehensive multidisciplinary approach to each patient is necessary and involvement of radiation oncologists, medical oncologists and urologic oncologists at the initial diagnosis may help to streamline appropriate and timely care for each patient.

## Conflict of Interest

None declared.
